# Empathy in Future Nurses: Insights for Healthcare Management from a Greek Student Sample

**DOI:** 10.3390/healthcare13162054

**Published:** 2025-08-20

**Authors:** Kejsi Ramollari, Nikolaos Kontodimopoulos

**Affiliations:** 1 School of Social Sciences, Hellenic Open University, 263 31 Patras, Greece; keisyramollari@gmail.com; 2 Department of Economics and Sustainable Development, Harokopio University, 176 76 Athens, Greece

**Keywords:** empathy, nursing education, healthcare management, student well-being, emotional workforce sustainability, professional development

## Abstract

**Background/Objectives:** Empathy is a core competency in nursing, contributing to patient care quality and professional resilience. This study investigated empathy levels among Greek undergraduate nursing students at the University of Peloponnese and examined the personal and educational factors that contribute to empathic development. **Methods:** A cross-sectional survey was conducted with 144 students from all academic years using the Jefferson Scale of Physician Empathy—Health Professions (JSPE-HP) and the SF-12 Health Survey. Data were analyzed using ANOVA and stepwise multiple linear regression. **Results:** Mean empathy scores were relatively high (M = 110.31, SD = 10.52). Empathy increased significantly with academic progression (*p* < 0.001), and higher scores were associated with parental status (*p* = 0.030) and better mental health (*p* = 0.044). Conversely, students with a chronically ill close contact reported lower empathy (*p* = 0.018). Regression analysis identified having children and exposure to chronic illness as significant predictors. **Conclusions:** Educational progression, life experience, and well-being are key contributors to empathy development. These insights support strategies to enhance empathy through curriculum design, student support, and wellness programs. Integrating empathy training into management policy can foster professional growth, reduce burnout, and improve patient care and workforce sustainability.

## 1. Introduction

Empathy, the ability to understand, share, and appropriately respond to the feelings of others, is fundamental to human social interactions. It includes cognitive, affective, and behavioral components. Cognitive empathy refers to recognizing another’s emotional state, affective empathy involves emotionally resonating with those feelings, and behavioral empathy is the capacity to express this understanding through compassionate and supportive actions, especially critical in healthcare contexts [1]. Empathy is essential in healthcare for several reasons. It forms the foundation of patient-centered care, which prioritizes the patient’s experience and individual needs, and enables healthcare professionals to build strong therapeutic relationships by fostering trust and open communication. This is crucial, as patients who trust healthcare providers are more likely to share relevant information, follow treatment plans, and actively engage in their care [2].

Empathy also contributes to greater diagnostic accuracy. When healthcare providers listen carefully and understand patients’ perspectives, they are better able to gather comprehensive information, leading to more accurate diagnoses and effective treatment plans [3]. Furthermore, empathy supports ethical medical practice by ensuring that patients are treated with dignity and respect, in accordance with fundamental principles of medical ethics [4].

Empathy plays a vital role in reducing burnout by enhancing interpersonal connections, job satisfaction, and emotional resilience. Burnout, which is marked by emotional exhaustion, depersonalization, and diminished personal accomplishment, is common among healthcare professionals. When properly supported, showing empathy can strengthen nurses’ sense of purpose and connection to their work, helping to protect them from burnout [5]. However, without good coping strategies, ongoing emotional stress may lead to empathy fatigue, highlighting the importance of support from institutions.

In addition, empathy improves communication and teamwork among healthcare professionals by fostering open and respectful dialogue, which is essential for collaboration [6]. It also enhances clinical competence through active listening, critical thinking, and effective communication, which are all fundamental skills for accurate diagnosis and treatment. Developing empathy, for nursing students, is an integral part of acquiring basic clinical skills as it sharpens observation and helps them to notice subtle cues in patient behavior and communication, which can lead to more accurate assessments and timely interventions [7]. Additionally, empathy encourages reflective practice, where students evaluate their interactions and decisions to improve their approach to care [8].

Quality of life, encompassing physical, psychological, social, and environmental well-being, is closely linked to empathy, particularly in emotionally demanding professions like nursing. Nursing students often face significant academic and clinical stressors that can impact their mental and physical health, potentially influencing their capacity for empathic engagement. Understanding and supporting their quality of life is therefore essential, not only for student well-being but also for sustaining empathy throughout their training and future clinical practice [9].

Recent literature highlights growing efforts to understand and cultivate empathy among nursing students, as it directly influences the quality of patient care and professional resilience. Meta-analyses and systematic reviews have shown that empathy levels tend to increase through targeted educational interventions, including simulation-based learning, reflective exercises, and communication training, with small to moderate effect sizes observed across studies [10,11]. A recent scoping review also emphasizes the role of experiential factors such as caregiving background, gender, and clinical exposure in shaping empathy during nursing education [12]. However, cultural and institutional differences can influence both empathy development and its measurement, underscoring the importance of localized studies. Despite global attention to this topic, there is limited empirical data from nursing programs in Greece.

This study investigates empathy levels specifically among nursing students, focusing on variables that reflect both their educational progression and life experiences relevant to caregiving. These include year of study, parental status, caregiving background, exposure to chronic illness, and self-reported physical and mental health. Such factors are particularly pertinent to the development of empathy within the context of nursing education and future professional practice. The findings not only contribute to nursing education but also offer valuable insights for healthcare management. Understanding how empathy develops, and what may hinder or enhance it, can help academic institutions and health systems implement targeted interventions that promote emotional intelligence, reduce burnout, and improve patient care quality. In this way, cultivating empathy in future nurses becomes not only an educational goal but also a strategic priority for healthcare organizations seeking to enhance staff well-being, team dynamics, and patient outcomes.

## 2. Materials and Methods

### 2.1. Sample and Data Collection

The research sample consisted of undergraduate nursing students enrolled across all academic years at the University of Peloponnese. This site was selected due to its accessibility and institutional collaboration. The nursing department follows a nationally standardized 4-year curriculum combining theory and clinical practice. Demographic characteristics of the student body, i.e., primarily female, aged 18–23, and from varied urban and rural backgrounds, are broadly representative of the national nursing student population.

This inclusive approach allowed for the assessment of empathy at various stages of nursing education, from early theoretical instruction to advanced clinical training. The total population of eligible students was approached during scheduled lectures and practical sessions, ensuring accessibility and minimizing disruption to their academic activities. These sessions were selected in collaboration with faculty and included core nursing courses across all academic years to ensure broad participation and representation of both classroom-based and clinical instruction.

Data collection was conducted in March 2024 using a paper-based questionnaire format. A total of 200 questionnaires were distributed by the researchers in coordination with faculty members, who facilitated access to classrooms and informed students about the voluntary and anonymous nature of participation. Students were given time to complete the survey on-site. Participation was voluntary, and informed consent was obtained from all respondents. Inclusion criteria required that students be currently enrolled in the nursing program and willing to participate. There were no exclusion criteria based on demographic characteristics.

### 2.2. Instruments

The instruments in this study were selected based on their good psychometric properties, relevance to the nursing student population, and availability in validated Greek versions. They offered a practical and well-established means of assessing empathy and health-related quality of life in the context of healthcare education.

#### 2.2.1. Jefferson Scale of Physician Empathy—Health Professions Version (JSPE-HP)

Empathy was assessed using the Jefferson Scale of Physician Empathy—Health Professions (JSPE-HP), a widely used tool for measuring empathy in healthcare professionals. The JSPE-HP consists of 20 items scored on a 7-point Likert scale ranging from 1 (strongly disagree) to 7 (strongly agree). Ten of the items (1, 2, 3, 4, 5, 6, 7, 13, 14, 15) are positively worded and scored directly. The remaining ten items (8, 9, 10, 11, 12, 16, 17, 18, 19, 20) are negatively worded and reverse-scored. The instrument evaluates three distinct factors: (i) perspective taking, i.e., the ability to delve into and analyze the patient’s problem “from outside”, (ii) “compassionate care”, and (iii) “standing in the patient’s shoes”.

Total scores range from 20 to 140, with higher scores indicating greater levels of empathy. The JSPE-HP aims to capture both the degree of emotional engagement and the level of understanding that healthcare professionals exhibit toward their patients [13,14,15]. The instrument has been translated into 56 languages and has been previously used in Greek studies [16]. It has demonstrated strong internal consistency, with Cronbach’s α values typically ranging from 0.80 to 0.89 [13,16]. In the current study, internal consistency was also high, with Cronbach’s α = 0.77.

#### 2.2.2. SF-12 Health Survey

Health-related quality of life was measured using the SF-12 questionnaire, which allows self-assessment of both physical (PCS) and mental (MCS) health components. The SF-12 is a shortened version of the SF-36 and includes questions covering eight dimensions of health. Four dimensions (physical function, physical role, emotional role, and mental health) are assessed with two items each, while the remaining four (bodily pain, general health, vitality, and social functioning) are assessed with one item each.

SF-12 scores are calculated using a weighting system based on data from the general US population and are reported as normalized T-scores, with a mean of 50 and a standard deviation of 10. This allows for international comparisons. The Greek version of the SF-12 has been tested in a representative sample of 1005 members of the Greek general population and has demonstrated good construct validity [17].

Τhe SF-12 has shown acceptable reliability for both physical (PCS) and mental (MCS) health components, with Cronbach’s α typically above 0.70 [18]. In our sample, Cronbach’s α was 0.47 for PCS-12 and 0.75 for MCS-12.

#### 2.2.3. Sociodemographic Questionnaire

A structured sociodemographic questionnaire was developed by the authors to collect background information relevant to empathy development in nursing students. It included items on age, gender, year of study, marital and parental status, employment, prior caregiving experience, personal or family history of chronic illness, and internship completion. This information was used to explore potential associations between individual characteristics and empathy scores.

### 2.3. Statistical Analyses

Descriptive statistics were calculated to summarize the sample’s demographic, academic, and personal characteristics, including means, standard deviations, and frequency distributions. To examine differences in empathy levels across subgroups, one-way analysis of variance (ANOVA) was applied. This method was used to test for statistically significant associations between empathy scores and variables such as year of study, gender, parental status, caregiving experience, presence of chronic illness (in oneself or in close contacts), employment status, and quality of life indicators.

For further analysis, physical and mental health scores were grouped into three predefined categories (tertiles): low, moderate, and high. This categorization allowed for a clearer comparison of empathy levels in relation to physical and mental health, as measured by the SF-12 Health Survey.

Using empathy (JES-HP score) as the dependent variable, multiple linear regressions were run to investigate the relationships with other sociodemographic and health-related variables. Parental status was originally coded as three categories (no children, one child, two children) but as the “one child” group contained only one respondent, it was recoded into a binary variable (parent vs. non-parent) for the regression analysis to ensure sufficient statistical power and stable estimates. Stepwise variable selection was used and R-squared was reported.

All analyses were performed using IBM SPSS Statistics software (version 28.0), and statistical significance was set at ≤0.05.

### 2.4. Ethical Issues

The study was approved by the Institutional Review Board (IRB) of the postgraduate program in Health Care Management at the Hellenic Open University (approval reference number: 161962/15 October 2023). The research was conducted in accordance with the ethical standards of the Declaration of Helsinki. All participants gave informed consent and were assured that participation was voluntary and could be withdrawn at any time.

## 3. Results

The study sample consisted of 144 nursing students, with a response rate of 72%, which reflects the majority of the available nursing student population at the university, providing sufficient variability across academic years and personal characteristics to explore associations of interest. Participants were predominantly women, representing all academic years and a range of geographic and educational backgrounds. Additional descriptive data showed that most participants were not employed at the time of the study, though some worked in healthcare or unrelated fields. A majority had not experienced caregiving responsibilities or the recent loss of a family member, while a smaller subset reported personal or close exposure to chronic illness. Among fourth-year students, only a small number had completed their internship. Complete details of these characteristics are provided in Table 1.

Empathy levels among nursing students were generally high, with a mean score of 110.31 (SD = 10.52), ranging from 76 to 132. Several demographic and experiential variables were examined, though many showed no statistically significant association with empathy. No significant differences were found based on gender (*p* = 0.336), age (*p* = 0.146), or childhood residence (urban, provincial, or rural; *p* = 0.534). Similarly, marital status was not associated with empathy levels (*p* = 0.199), with mean scores ranging from 107.83 in students cohabiting with a partner to 116.55 in married students.

Academic background and employment status also showed no statistically significant effects. Students with an additional degree had slightly higher scores (113.79 vs. 109.94; *p* = 0.194), and those working in nursing roles reported higher scores (117.73) than those employed in unrelated fields (108.97), though these differences were not statistically significant (*p* = 0.069). Other personal experiences, including the loss of a close relative (*p* = 0.182), prior caregiving (*p* = 0.721), and the presence of chronic illness (*p* = 0.428), were also not significantly associated with empathy. Also, completion of a clinical internship did not appear to influence empathy levels (*p* = 0.954).

To facilitate group comparisons, physical (PCS-12) and mental (MCS-12) health scores were divided into tertiles, creating three equally sized groups: low (1st tertile), moderate (2nd tertile), and high (3rd tertile) levels. Physical health was also not significantly associated with empathy (*p* = 0.625).

In contrast, several variables demonstrated statistically significant associations with empathy, reflecting meaningful differences between groups. Detailed ANOVA results for these comparisons are presented in Table 2.

The analysis based on the year of attendance showed that average empathy scores increased progressively, ranging from 103.54 in the first year to 116.50 in the fifth year and above. Second-year students had an average score of 114.51, third-year students 110.73, and fourth-year students 110.03. ANOVA analysis revealed a statistically significant difference (*p*-value < 0.001) in empathy levels across different years of study.

Empathy scores significantly differed according to parental status (*p*-value = 0.030), with average scores ranging from 109.76 for students without children to 123.00 for those with one child and 119.29 for those with two or more children. Furthermore, having someone in one’s immediate social circle with a long-term medical condition shows a statistically significant effect on empathy (*p*-value = 0.018). Average empathy scores were 111.71 for students without such exposure, compared to 107.24 for those who had a close contact with chronic illness, suggesting a potential association between this experience and lower empathy scores.

ANOVA revealed significant differences in empathy scores across MCS-12 tertiles. Students with low, moderate, and high mental health levels had mean empathy scores of 110.60, 107.50, and 112.83, respectively (*p* = 0.044). These findings suggest that higher mental health is associated with greater empathic capacity, highlighting a positive link between emotional well-being and empathy in nursing students.

To evaluate the relative contribution of factors significantly associated with empathy, a multiple linear regression analysis was conducted using a stepwise variable inclusion approach. Variables entered into the model included year of study, parental status, chronic illness in a close contact, and mental health (MCS-12). Gender, age, and physical health (PCS-12) were initially considered but were excluded from the final model for parsimony, as they did not meaningfully affect the results. Only variables that remained statistically significant appear in Table 3.

The stepwise multiple linear regression model identified two significant predictors of empathy scores: having children and having a close contact with a chronic illness. Students with children had significantly higher empathy scores (unstandardized β = 9.195, *p* = 0.015), while those with a close contact with chronic illness had slightly lower empathy scores (unstandardized β = −4.017, *p* = 0.030). The overall model accounted for 6.6% of the variance in empathy scores (R^2^ = 0.066).

## 4. Discussion

This study aimed to explore empathy levels among undergraduate nursing students and identify personal and educational factors associated with its development. Using a cross-sectional design, we administered the Jefferson Scale of Physician Empathy—Health Professions version (JSPE-HP) and the SF-12 Health Survey to a sample of 144 students across all academic years. Our findings suggest that empathy increases as students progress through their nursing education, supporting the role of clinical exposure and reflective learning in promoting empathic growth. This aligns with prior studies showing that structured clinical engagement enhances emotional awareness and interpersonal skills [10].

### 4.1. Comparison of Findings with Literature

The overall empathy score in our sample (M = 110.31) is comparable to that reported in similar populations using the JSPE-HP. For instance, Aranha et al. [19] reported mean scores of 104 among South African nursing students, while Huang et al. [20] found an average of 103 in Chinese nursing and midwifery students. Both studies, like ours, observed higher empathy levels in students further along in their programs. Our results fall within the upper international range, possibly reflecting features of Greek nursing education such as an emphasis on patient-centered care and culturally embedded caregiving values [21]. The increase in empathy across academic years also supports previous findings that highlight the contribution of clinical exposure and reflective learning to empathic development. Evidence from recent meta-analyses demonstrates that simulation-based educational interventions lead to meaningful improvements in student empathy, particularly in programs implemented after 2019 [22].

Our results are also in line with Williams et al. [23], who found significantly higher empathy scores in third-year compared to first-year Australian nursing students, attributing the increase to the cumulative impact of nursing education and clinical practice. This likely reflects core components of the curriculum, including progressive clinical exposure and structured reflection, which cultivate empathy as a professional competency. Similarly, international research using the JSPE-HP confirms a pattern of rising empathy with educational advancement [24]. However, contrasting evidence from Ward et al. [25] showed a decline in empathy over time, which was attributed to academic pressure and burnout. Additionally, other studies have reported no clear association between academic progress and empathy [26].

Empathy-centered teaching, such as disabled health courses and transformative learning modules, has shown promise in promoting empathy, though effects vary across populations [27]. Furthermore, community-engaged education not only cultivates empathy but also reinforces students’ sense of social accountability and professional identity. Overall, empathy development is complex in nature [28]. A scoping review of educational interventions in nursing reinforces that, while most programs enhance empathy, variability in methods and outcomes highlights a need for standardized tools and designs [29].

### 4.2. Interpretation of the Results

In this study, parental status was a variable associated with empathy, supporting previous research that links parenting and informal caregiving to greater emotional attunement and perspective taking [23]. Similarly, Plank et al. [30] found that mothers exhibited higher empathic responsiveness, suggesting that parenthood may enhance overall emotional sensitivity rather than empathy limited to interactions with children. In contrast, students with close contacts suffering from chronic illness showed lower empathy levels. This may be due to emotional strain or early signs of compassion fatigue, which is consistent with prior research on caregiver burden [31,32].

The observed reduction in empathy among students with chronically ill contacts adds nuance to this relationship. While caregiving can increase understanding of patient experiences, it may also lead to emotional exhaustion, especially when sustained over time [33]. This condition, known as empathy fatigue or compassion fatigue, results in diminished capacity for empathy due to prolonged exposure to others’ suffering [32]. Informal caregivers are particularly susceptible due to the psychological and emotional demands involved [31].

No significant association was found between empathy and physical health scores. Although previous studies have suggested links between high empathy and physical symptoms such as headaches, gastrointestinal issues, and sleep disturbances, these were not evident in our data. Such symptoms have been attributed to the mental demands of empathic engagement [34], and chronic stress related to empathy may elevate cortisol levels, impairing immune function [35]. The lack of association in our findings may be due to sample-specific coping mechanisms or differing levels of clinical stress. Further investigation is warranted to clarify this complex relationship in nursing students.

In contrast, higher empathy was positively associated with better mental health. Students with elevated empathy reported improved mental health outcomes, reinforcing earlier studies that link empathy with reduced anxiety and depression. Thomas et al. [36], for example, found that empathetic nursing students experienced fewer mental health challenges. These results suggest that empathy may serve a protective role against common psychological stressors in healthcare education and practice.

In this study, the regression analysis identified two significant predictors of empathy: having children and having a close contact with a chronic illness. Students with children showed higher empathy, likely due to the emotional demands of parenthood enhancing perspective taking. In contrast, those with a chronically ill contact had slightly lower empathy, possibly reflecting emotional fatigue or stress. The model explained 6.6% of the variance in empathy scores, which is reasonable given the complex, multifactorial nature of empathy. This modest explanatory power underscores the need for future research to examine additional influences such as personality traits, cultural context, and clinical learning environments, in order to more fully understand empathy development in nursing students.

### 4.3. Strengths and Limitations

This study has several strengths. It contributes new evidence on empathy development among nursing students in Greece, a population rarely examined in the international literature. By including students from all four academic years, the study provides insights into how empathy evolves across the educational trajectory, which can inform curriculum design. The use of two well-established, psychometrically sound instruments (JSPE-HP and SF-12) strengthens the reliability and comparability of the findings with international studies. Finally, the study goes beyond descriptive analysis by using multivariable regression to explore the relative contribution of different factors, adding depth to the understanding of empathy development.

This study has some limitations as well. Its cross-sectional design precludes causal interpretations of the relationships identified. Longitudinal studies are needed to examine how empathy develops over time, as well as to assess the impact of specific educational interventions. Additionally, the sample was drawn from a single university using convenience sampling, limiting the generalizability of the results. Future research should consider incorporating qualitative or mixed-methods designs to capture deeper insights into empathy development.

### 4.4. Practical Implications for Education and Management

The findings of this study have several practical implications for both nursing education and healthcare management. The observed increase in empathy across academic years underscores the importance of integrating clinical exposure and reflective practice early and consistently in the nursing curriculum [37]. Incorporating experiential learning that engages students with real-life caregiving situations may further support empathic development. Additionally, the positive association between empathy and mental well-being highlights the value of wellness initiatives, such as mentoring, counseling, and stress management programs, in promoting emotional resilience. These educational strategies are not only essential for preparing students to deliver high-quality, compassionate care but also contribute to reducing burnout and enhancing teamwork in clinical settings [38,39].

From a healthcare management perspective, the results point to actionable strategies that strengthen both training and workforce development. Institutional policies that promote well-being among students and staff such as mindfulness programs, structured reflection, and mentoring can help sustain empathy while mitigating emotional fatigue. Recognizing that empathy is shaped by both educational experiences and personal circumstances emphasizes the need for flexible, individualized approaches to training [40]. By integrating these elements into nursing education and staff development, healthcare leaders can cultivate a more emotionally intelligent and resilient workforce, leading to improved patient satisfaction, stronger team dynamics, and better staff retention [41].

## 5. Conclusions

The educational journey in nursing combines theoretical learning with practical experience. Early academic years focus on foundational knowledge, while later stages emphasize clinical practice and patient interaction. This progression enables students to apply theory in real-world contexts, deepening their understanding and empathy. Ongoing patient engagement and reflective practice foster a more nuanced awareness of patient needs and experiences.

Based on our findings, we recommend that nursing education programs incorporate structured empathy training throughout the curriculum, particularly in the early years, to support consistent development. The positive association between empathy and mental health also supports the inclusion of wellness and reflective components that build emotional resilience. Life experiences such as caregiving and parenthood further influence empathy, highlighting the importance of personal reflection and shared experience in both academic and clinical settings.

These insights also offer practical direction for healthcare management. Nursing schools and institutions should embed empathy training, reflective practices, and wellness support into both education and workplace environments. The influence of personal factors such as mental health, caregiving, and parental status underscores the need for flexible, individualized approaches. As a strategic priority, cultivating empathy enhances patient care, promotes staff well-being, and contributes to a more resilient healthcare workforce.

## Figures and Tables

**Table 1:** Characteristics of the sample.

Demographic/Educational Background	Ν (%)	Life Experience/Personal Circumstances	Ν (%)
Gender		Loss of a family member	
Male	24 (16.67)	Yes	81 (56.25)
Female	119 (82.64)	No	63 (43.75)
Other	1 (0.69)	Prior experience as a caregiver	
Age		No	94 (65.28)
18–20	62 (43.05)	Yes, to a relative	44 (30.56)
21–23	66 (45.83)	Yes, to a child with special needs	0 (0)
24–26	5 (3.47)	Yes, other	6 (4.17)
27+	11 (7.59)	Chronic illness of the person cared for	
Year of study		Yes	20 (13.89)
First	28 (19.44)	No	124 (86.11)
Second	37 (25.69)	Chronic illness of a close contact	
Third	44 (30.56)	Yes	45 (31.25)
Fourth	31 (21.53)	No	99 (68.75)
Fifth or above	4 (2.78)	Completed internships for 4th year students	
Place of origin		Yes	10 (6.94)
Large urban center	71 (49.31)	No	25 (17.36)
Provincial town	49 (34.03)	Not applicable	109 (75.69)
Village	24 (16.67)	Parental status	
Family status		No children	136 (94.44)
Unmarried	123 (85.42)	Yes, one child	1 (0.69)
Married	11 (7.64)	Yes, two or more children	7 (4.86)
Cohabiting with significant other	6 (4.17)		
No answer	4 (2.78)		
Other degree			
Yes	14 (9.72)		
No	130 (90.28)		
Work			
No	98 (68.06)		
Yes, as nurse	11 (7.64)		
Yes, other healthcare profession	3 (2.08)		
Yes, other	32 (22.22)		

**Table 2:** ANOVA Results for variables significantly associated with empathy scores.

Variable	JES-HP Means	F-Value	df (Between/Within)	*p*-Value
Year of study				
First	103.54	4/139	5.317	<0.001
Second	114.51			
Third	110.73			
Fourth	110.03			
Fifth or above	116.50			
Parental status				
No children	109.76	2/141	3.585	0.030
One child	123.00			
Τwo or more children	119.29			
Chronic illness of a close contact				
Yes	107.24	1/142	5.747	0.018
No	111.71			
MCS-12				
1st tertile (lower mental health)	110.60	2/141	3.205	0.044
2nd tertile (moderate mental health)	107.50			
3rd tertile (high mental health)	112.83			

**Table 3:** Results of stepwise multiple linear regression predicting empathy scores.

Variables	Coefficients		Sig.	R^2^
	Unstd. Beta	Std. Error		
Constant	115.074	2.584	<0.001	0.066
Children (yes)	9.195	3.719	0.015	
Chronic illness of a close contact	−4.017	1.838	0.030	

Note: Only significant (*p* < 0.05) predictor variables are presented.

## Data Availability

Data is available from the authors upon request.
